# Pathogenic KCNH2-G53S variant in the PAS domain influences the electrophysiological phenotype in long QT syndrome type 2

**DOI:** 10.3389/fcvm.2025.1524909

**Published:** 2025-04-09

**Authors:** Dasom Mun, Ji-Young Kang, Malgeum Park, Gyeongseo Yoo, Nuri Yun, YouMi Hwang, Boyoung Joung

**Affiliations:** ^1^ Division of Cardiology, Yonsei University College of Medicine, Seoul, Republic of Korea; ^2^ GNTPharma Science and Technology Center for Health, Incheon, Republic of Korea; ^3^ Division of Cardiology, Department of Internal Medicine, St. Vincent’s Hospital, The Catholic University College of Medicine, Suwon, Republic of Korea; ^4^ Catholic Research Institute for Intractable Cardiovascular Disease (CRID), College of Medicine, The Catholic University of Korea, Seoul, Republic of Korea

**Keywords:** long QT syndrome type 2, KCNH2^G53S^, PAS domain, variant, hiPSC-CMs

## Abstract

**Background:**

Long QT syndrome type 2 (LQT2) is an arrythmia caused by loss-of-function mutations in KCNH2, leading to impaired Kv11.1 channel function.

**Objective:**

To better understand LQT2, we examined the electrophysiological differences related to the G53S variant, which is located within the PAS domain of KCNH2, using patient-specific human induced pluripotent stem cell (hiPSC)-derived cardiomyocytes (hiPSC-CMs).

**Methods:**

We generated hiPSC-CMs from a patient harboring the KCNH2^G53S^ variant and a healthy control using non-integrative Sendai virus-mediated reprogramming. Their electrophysiological properties were assessed using microelectrode arrays (MEA), and Ca^2+^ dynamics were characterized using Fluo-4 dye.

**Results:**

The patient harboring KCNH2^G53S^ experienced aborted sudden cardiac death at 22 years of age, was diagnosed with LQT, and had an implantable cardioverter-defibrillator (ICD) implanted. KCNH2^G53S^ hiPSC-CMs expressed less KCNH2 than normal CMs. Transcriptomic analysis of KCNH2^G53S^ hiPSC-CMs revealed 3,857 differentially expressed genes, highlighting significant changes in pathways related to LQT2 development. Action potential duration was significantly longer in KCNH2^G53S^ hiPSC-CMs than in control (545.3 ± 176.3 ms vs. 339.9 ± 44.5 ms; *P =* 0.019). Corrected field potential duration was significantly longer in KCNH2^G53S^ hiPSC-CMs than in control (318.0 ± 66.3 ms vs. 234.5 ± 21.0 ms; *P =* 0.015), indicating altered electrophysiology. KCNH2^G53S^ hiPSC-CMs exhibited significantly increased calcium transient amplitude and prolonged calcium wave duration under isoproterenol stimulation, indicating exacerbated abnormal calcium handling.

**Conclusion:**

Our analysis of hiPSC-CMs carrying a heterozygous KCNH2^G53S^ mutation, which showed abnormal electrophysiology and impaired calcium handling, provides a basis for developing improved management strategies for patients with LQT2.

## Introduction

Long QT syndrome (LQT) is an inherited arrhythmia that leads to QT interval prolongation, syncope, and sudden cardiac death (SCD) due to ventricular tachyarrhythmia (1–3). Congenital LQT is caused by a mutation in one of several genes that encode cardiac ion channels (4). Approximately 90% of LQT cases are linked to mutations in the KCNQ1 (type 1 LQT; LQT1), KCNH2 (type 2 LQT; LQT2), and SCN5A (type 3 LQT; LQT3) genes, and about 45% of these cases are caused by changes in the potassium channels KCNQ1 and KCNH2 (5, 6). Pathogenic variants in the KCNH2 gene, which encodes the α-subunit of the hERG channel, cause LQT2 (7). More than 700 rare sequence variants in the KCNH2 gene, the majority of which are loss-of-function mutations, impair the delivery of Kv11.1 proteins to the cell membrane, contributing to a significant proportion of LQT cases (8). The N-terminal Per/Arnt/Sim (PAS) domain is a well-established hotspot for pathogenic variants (9, 10), playing a crucial role in channel interactions and the slow deactivation of the hERG channel (11, 12). Given its functional importance, investigating PAS domain mutations is essential for understanding their impact on hERG channel function. Patient-specific hiPSC-derived cardiomyocytes (hiPSC-CMs) carrying KCNH2 mutations serve as an effective model for evaluating the cellular and physiological consequences of pathogenic variants. Previous studies have demonstrated that LQT2-hiPSC-CMs exhibit prolonged action potentials, reduced IKr, and early afterdepolarizations (EADs), mirroring the electrophysiological defects observed in LQT patients (13, 14). The derived cardiomyocytes had significantly prolonged action potential, diminished IKr, and triggered arrhythmias. However, because resources to study disease-specific mutations in KCNH2 *in vitro* are lacking, patient-specific cardiomyocytes with mutations in KCNH2 are needed.

In this study, we established a human induced pluripotent stem cell (hiPSC) line from the peripheral blood monocytes (PBMCs) of a patient with LQT2 who carries a pathogenic KCNH2 variant (c.157G>A; p.Gly53Ser) within the PAS domain. We then generated a hiPSC-derived cardiomyocyte (hiPSC-CM) line to investigate the mechanism by which this pathogenic variant in the PAS domain causes LQT2. Using these model cardiomyocytes, we aimed to study the mechanism by which pathogenic mutations in the PAS domain of KCNH2 lead to LQT.

## Methods

### Ethical approval

All studies were approved by the Institutional Review Board (IRB) of Severance Hospital of Yonsei University (IRB No. 4-2022-1012), and signed informed consent was obtained from the donor. The human stem cell line, CMC-hiPSC-011 cells were obtained from the Korean National Stem Cell Bank.

### Isolation of PBMCs and reprogramming to hiPSCs

Blood samples were collected in K2-EDTA tubes (BD Vacutainer), and PBMCs were isolated by gradient centrifugation using Lymphoprep™ Density Gradient Medium (STEMCELL Technologies). After isolation, 2–4 × 10^6^ cells were cryopreserved in 10% DMSO and 90% FBS until reprogramming. Then, 1 × 10^6^ of the PBMCs were plated in StemSpan™ SFEM II medium (STEMCELL Technologies; cat#09605) supplemented with cytokines (StemSpan™ Erythroid Expansion Supplement; STEMCELL Technologies; cat#02692). Somatic reprogramming was performed using the Epi5™ Episomal hiPSCs Reprogramming Kit using electroporation (Neon transfection system; Thermo Fisher Scientific) according to the manufacturer's recommendations. Approximately of 1.0 × 10^7^ cells/ml were electroporated with 1ul of Epi5™ reprogramming vectors and 1 μl of Epi5™ p53 & EBNA vectors under the condition of 1,650 V, 10 ms, and 3 pulses by Neon™ Transfection System (Thermo Fisher Scientific). The iPSC colonies typically arise between days 18–25, were picked up, and subjected to PCR amplification and sequencing.

The hiPSCs were maintained in feeder-free conditions on Matrigel®-coated plates (Corning) in TeSR™ medium (StemCell Technologies) at 37°C and 5% CO_2_ and were subcultured every 4–6 days with 10 μM Y27632 (Sigma). The hiPSCs were maintained in TeSR™ medium (STEMCELL Technologies) on Matrigel®-coated plates (Corning) at 37°C and 5% CO_2_ and were subcultured every 4–6 days with 10 μM Y27632 (Sigma).

### Sanger sequencing

Genomic DNA was extracted using the G-spin^TM^ Total DNA Extraction Mini Kit (iNtRON Biotechnology). The DNA fragment around the knock-in region was amplified by PCR using AccuPower® PCR PreMix (BIONEER). The PCR products were sequenced using Sanger sequencing at BIONICS Co., Ltd. (Korea). Sequencing data were analyzed using SnapGene software (GSL Biotech; available at snapgene.com).

### Immunocytochemistry

Cells were fixed with 4% paraformaldehyde for 15 min and permeabilized with 0.3% Triton-X100 for 30 min at room temperature (RT). After blocking with 1% BSA for 30 min at RT, the cells were incubated with the following primary antibodies: anti-MLC2 V (1:200, Santa Cruz Biotechnology, sc-517244), anti-cTnI (1:200, Santa Cruz Biotechnology, sc-133117), anti-Nkx 2.5 (1:200, Santa Cruz Biotechnology, sc-376565), anti-MLC2A (1:200, Santa Cruz Biotechnology, sc-365255), anti-hERG (1:200, Santa Cruz Biotechnology, sc-377388), and anti-α-actinin (1:200, Santa Cruz Biotechnology, sc-17829) overnight at 4°C. Then, the cells were incubated with a fluorescence-conjugated secondary antibody. The cells were washed with PBS, and the nuclei were stained with Hoechst 33342 (Thermo Fisher Scientific). The stained cells were visualized using a confocal microscope (Zeiss LSM 710).

### Differentiation of hiPSC-CMs

The hiPSCs derived from the patient with the KCNH2 mutation were differentiated into ventricular cardiomyocytes using a monolayer-based small-molecule protocol as described previously (15), with some modifications. The hiPSCs were maintained in TeSR™ medium on Matrigel®-coated plates, dissociated into single cells, and then seeded onto Matrigel®-coated 6-well plates. The differentiation started with changing TeSR™ medium to RPMI + B27 supplement without insulin, supplemented with 4 μM CHIR99021 (Tocris Bioscience). After 24 h, the medium was changed to RPMI + B27 without insulin, and the cells were cultured in this medium for 48 h. On day 3, cells were treated with 2 μM IWP2 (Tocris Bioscience) in 3 ml of fresh RPMI + B27 without insulin for 48 h. On day 5, the medium was changed to RPMI + B27 without insulin; on day 7, the medium was changed to RPMI + B27 complete supplement (with insulin). The differentiated cells were maintained in this medium until day 15, and the medium was changed every other day. On day 15, the differentiated hiPSC-CMs were purified using lactate medium (RPMI without glucose) + B27 supplement and incubated for 10 days. Then, the hiPSC-CMs were maintained in RPMI + B27 supplement until day 30. On day 30, the differentiated hiPSC-CMs were replated onto 35-mm glass-bottom culture dishes for imaging, optical measurement of Ca^2+^ transients.

### Quantitative reverse transcription PCR (qRT-PCR)

Total RNA was extracted from the samples using the miRNeasy Mini Kit (Qiagen, Hilden, Germany) according to the manufacturer's instructions. RNA was reverse transcribed to complementary DNA (cDNA) using the High-capacity cDNA Reverse Transcription Kit (Applied Biosystems, Darmstadt, Germany). mRNA expressions were performed using QuantStudio 3 Real-Time PCR System (Applied Biosystems, USA) and PowerUp™ SYBR™ Green Master Mix (Applied Biosystems, USA). PCR primers were synthesized by Cosmo Genetech (Daejeon, Korea) and are listed in Supplementary Table S1. Relative mRNA expression was calculated using the 2^−ΔΔCq^ method and normalized against the expression level of β-actin.

### RNA sequencing

RNA sequencing was performed by Macrogen (Daejeon, Korea). Library preparation was performed using the TruSeq Stranded mRNA Kit (Illumina, San Diego, CA, USA) according to the manufacturer's protocol. The libraries were sequenced on the Illumina NovaSeq 5000 instrument (Illumina). The R package edgeR was used for data normalization. The heatmap and hierarchical clustering plot were provided using the pheatmap package in R. GO pathway analysis was performed using the ClueGO package in Cytoscape. Enrichment of KEGG pathways was analyzed by clusterProfiler version 3.16.1 and the pathview R package. Functional enrichment was performed using the g:Profiler web server (http://bitt.cs.ut.ee/gprofiler/) (16).

### Multi-electrode array

The hiPSC-CMs (5.0 × 10^4^) were seed into a Cytoview MEA 24-well plate (Maestro Multiwell MEA System, Maestro edge; Axion BioSystems Inc.; Atlanta, GA, USA) coated with 50 μg/ml fibronectin (Sigma-Aldrich). The medium was changed every 2 days until the electrophysiological signals of the CMs appeared. The MEA plates seeded with hiPSC-CMs were transferred directly from the incubator to the Maestro Multiwell MEA System (Axion BioSystems). Spontaneously beating human iPSC-CM electrophysiological activities, including field potential duration (FPD), beat period, spike amplitude, spontaneous beating rate, and action potential duration, were recorded using a microelectrode array (MEA) system (Maestro, Axion Biosystems). Recordings were taken for 5 min every 25 min following drug treatment using AxIS Navigator 2.0.4 (Axion Biosystems). MEA data were subsequently analyzed using the AxIS Metric Plotting Tool 2.2.5 (Axion Biosystems).

### Measurement of Ca^2+^ transients

The hiPSC-CMs were cultured on Matrigel®-coated cover glass slides and loaded with 2 μM Ca^2+^-sensitive Fluo-4-AM dye (Invitrogen, Carlsbad, CA, USA) for 20 min. After washing with PBS, the medium was replaced with live-cell imaging solution (A14291DJ; Invitrogen). To measure calcium transients, Ca^2+^-loaded cardiomyocytes were excited at 488 nm, and emissions were visually recorded at 500–550 nm using a 20× objective and a confocal microscope (Zeiss LSM 710; Carl Zeiss). To determine arrhythmogenic Ca^2+^ waves, longitudinal line scans were obtained using a confocal microscope (Bio-Rad) equipped with a Radiance 2100 40× oil immersion objective in line scan mode (3 ms/line). To quantify calcium transients, fluorescence intensity was calculated using *Δ*F/F₀, where F₀ represents the baseline fluorescence intensity before transient initiation, and F_peak_ is the maximum fluorescence intensity during the transient. The normalized fluorescence ratio was determined using the formula: ΔF/F₀ = F_peak_ − F_0_/F_0_.

### Statistical analyses

Statistical analysis between two groups was performed using a two-tailed Student's t-test, and more than two groups were analyzed using the one-way analysis of variance, followed by Tukey's *post hoc* test. A *p*-value less than 0.05 was considered statistically significant. All statistical analyses were performed using GraphPad Prism software version 8.0 (San Diego, CA, USA). Data are shown as the mean ± standard deviation unless otherwise stated.

## Results

### Patient description

**Figure 1:**
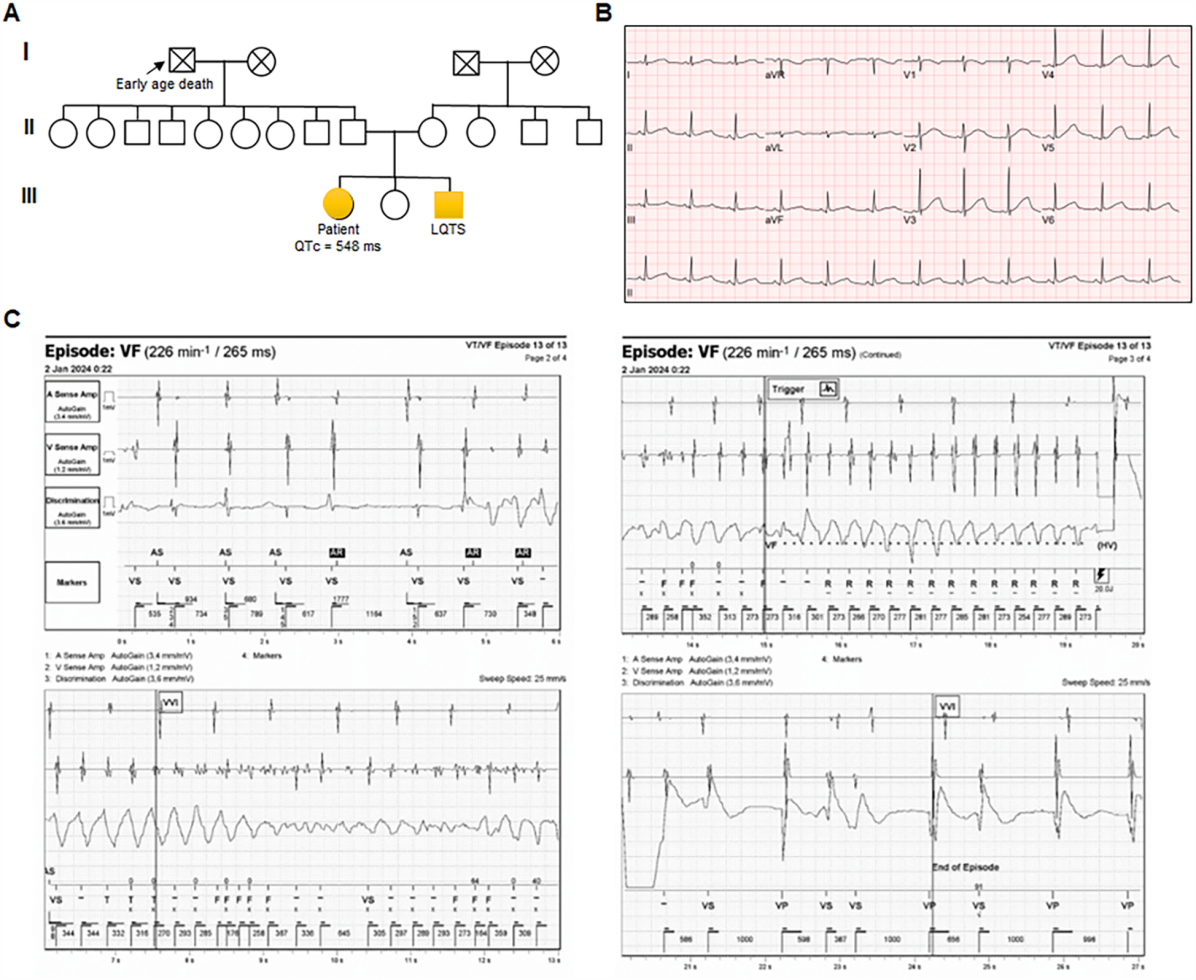
Characterization of the patient carrying KCNH2 p.G53S **(A)** pedigree of a family with autosomal dominant genetically elusive long QT syndrome (LQT). Squares and circles indicate male and female family members, respectively. Solid symbols indicate the presence of LQT. **(B)** Representative electrocardiogram (ECG) from the index case (QTc = 548 ms). **(C)** Episodes of ventricular fibrillation (VF) captured via implantable cardioverter-defibrillator (ICD) monitoring.

The proband with LQT was a highly symptomatic 36-year-old woman with a history of sudden syncope and seizures. Pedigree analysis identified three additional affected family members, including a grandfather, who died suddenly at a young age (Figure 1A). The first episode of concern, aborted sudden cardiac death, occurred at 22 years of age. She was then diagnosed with LQT, and an implantable cardioverter-defibrillator (ICD) was implanted at another institution. Resting ECG showed a corrected QT interval (QTc) of 548 ms (Figure 1B). Because she had mental stress and poor compliance with daily atenolol (50 mg), she experienced multiple ventricular tachycardia (VT) storms [due to VT and ventricular fibrillation (VF)] that were terminated by ICD shocks. Although she had undergone left sympathetic denervation and was adherent to daily doses of bisoprolol (5 mg) and flecainide (200 mg) with potassium supplementation (1,800 mg), she recently experienced ICD shocks due to VF after stress (Figure 1C). After changing flecainide to daily propafenone (450 mg), the VF events were controlled.

### Mutation validation of patient-specific hiPSC-CMs

**Figure 2:**
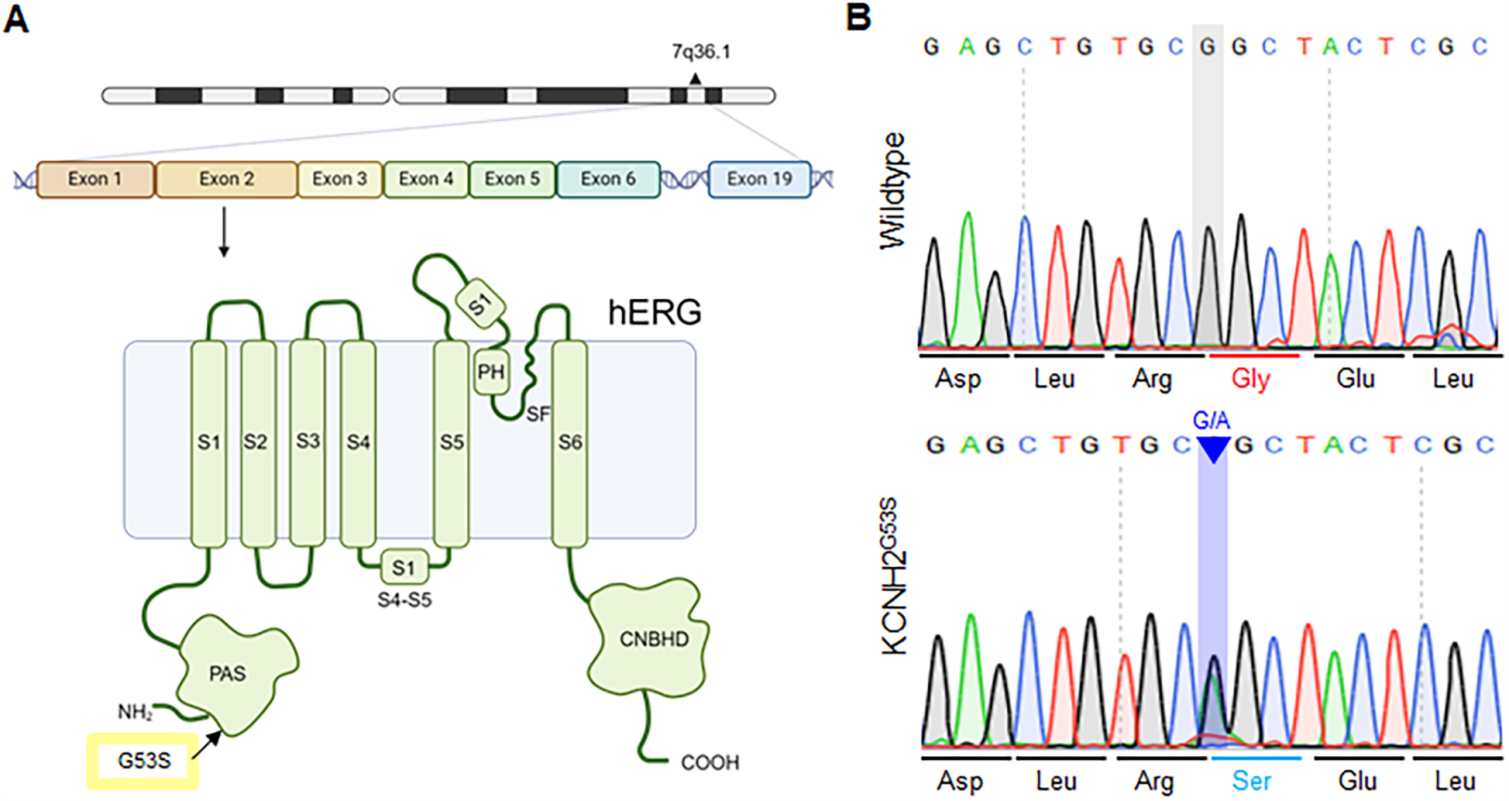
Schematic of the KCNH p.G53S mutation within the PAS domain **(A)** schematic representation of the c.157G>A mutation, located within the exon 2 region of KCNH2, and the location of p.G53S in the PAS domain of the hERG channel. **(B)** Sanger sequencing of the KCNH2^G53S^ hiPSC line showing that it harbors the heterozygous mutation c.157G>A in the KCNH2 gene.

Genetic analysis of the patient revealed a novel heterozygous mutation, c.157G>A, in KCNH2, which encodes the a-subunit of the hERG cardiac potassium channel. The KCNH2 protein consists of a cytoplasmic N-terminus, six α-helical transmembrane segments (S1–S6), interdomain links, extracellular P-loops, and a cytoplasmic C-terminus (17). S1–S4 are the primary voltage sensors for channel opening, whereas S5 and S6 are involved ion pore formation (18). Schematic representation of the mutation site (p. G53S), which is encoded within exon 2 of KCNH2, in the PAS domain of the hERG channel (Figure 2A). To evaluate the impact of the G53S variant, we generated hiPSCs from the patient's peripheral blood mononuclear cells (PBMCs) using Sendai virus-based reprogramming. Sanger sequencing confirmed that the derived hiPSC line harbored a heterozygous mutation (c.157G>A) resulting in a Gly53-to-Ser substitution in the PAS domain of KCNH2 (Figure. 2B). Taken together, a novel heterogeneous KCNH2^G53S^ mutation was identified in the patient with LQT2, and the model hiPSC line harboring this mutation is a valuable platform for studying the pathogenesis of LQTs.

### Generation and characterization of patient-specific hiPSC-CMs

**Figure 3:**
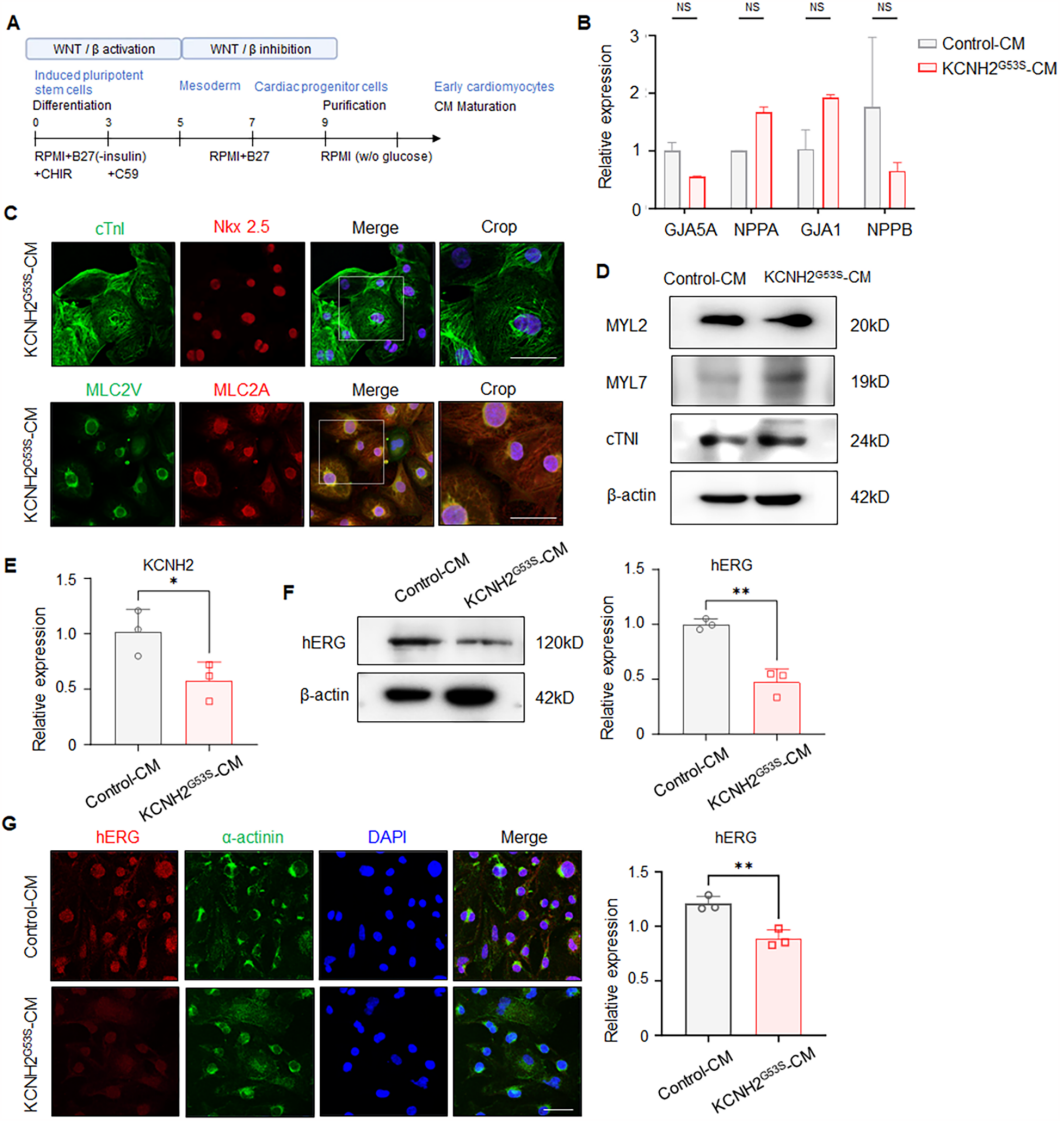
Generation of patient-specific induced pluripotent stem cell-derived cardiomyocytes (hiPSC-CMs) **(A)** detailed protocol for differentiating pluripotent stem cells into ventricular cardiomyocytes. **(B)** qRT-PCR to detect GJA5A, NPPA, GJA1, and NPPB in control and KCNH2^G53S^ hiPSC-CMs. **(C)** Representative immunostaining for NKX2.5, cardiac troponin I (cTNI), MLC2v, and MLC2a in control and KCNH2^G53S^ hiPSC-CMs. Nuclei are stained with DAPI (blue). Scale bar, 100 µm. **(D)** Western blotting and quantification of MYL2, MYL7, and cardiac troponin I (cTNI). β-Actin is a loading control. **(E)** qRT-PCR of KCNH2 mRNA expression in control and KCNH2^G53S^ hiPSC-CMs. **(F)** Western blotting and quantification of hERG in control and KCNH2^G53S^ hiPSC-CMs. β-Actin is a loading control. **(G)** Immunofluorescence staining of control and KCNH2^G53S^ hiPSC-CMs. Nuclei are stained with DAPI (blue). Scale bar, 50 µm. Data are the mean ± SD. *P < 0.05, **P < 0.01.

To evaluate the effect of the KCNH2^G53S^ variant, we differentiated patient-derived hiPSCs into cardiomyocytes (hiPSC-CMs) using a chemical protocol with a B27 supplement minus insulin (Figure 3A). qRT-PCR analysis revealed that the generated hiPSC-CMs expressed the cardiomyocyte-specific markers GJA5A, NPPA, GJA1, and NPPB (Figure 3B). Additionally, immunostaining for the cardiac proteins NKX2.5, cardiac troponin I (cTNI), MLC2v, and MLC2a was performed on both control and KCNH2^G53S^ hiPSC-CMs to verify their presence and confirm their identity as cardiomyocytes (Figure 3C). We further examined the expression levels of a ventricle marker (MYL2) and an atrial marker (MYL7) using western blot analysis, confirming that both the control hiPSC-CMs and KCNH2^G53S^ hiPSC-CMs were differentiated into ventricle cells, confirming their suitability for studying the effects of the KCNH2 G35S mutation (Figure 3D).

We then characterized these patient-specific hiPSC-CMs to understand the effects of the G35S variant on hERG expression and localization by using qRT-PCR, western blotting, and immunofluorescence staining. qRT-PCR analyses showed that KCNH2 expression was lower in the KCNH2^G53S^ hiPSC-CMs than in the control hiPSC-CMs (Figure 3E). Western blot analysis showed that hERG expression was lower in the KCNH2 G53S mutant hiPSC-CMs than in control hiPSC-CMs (Figure 3F). Immunofluorescence staining indicated that hERG expression was lower in KCNH2^G53S^ hiPSC-CMs than in control hiPSC-CMs (Figure. 3G). These results showed that the mutation caused a defect in hERG expression.

### Differentially expressed genes of KCNH2^G53S^ hiPSC-CMs

**Figure 4:**
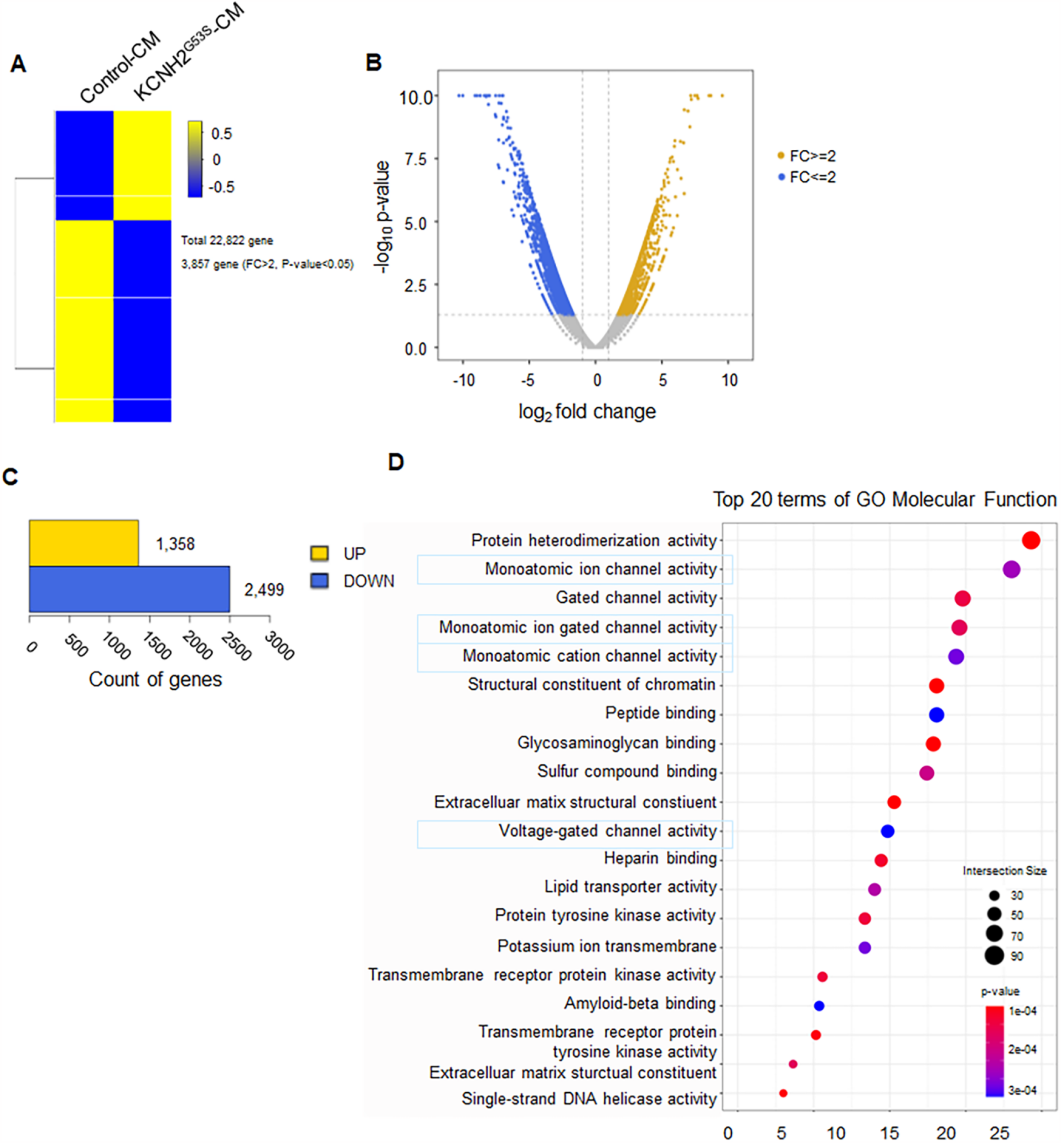
RNA sequencing of the transcriptome of KCNH2^G53S^ hiPSC-CMs **(A)** heatmap of the differentially expressed genes (DEGs) in control and KCNH2^G53S^ hiPSC-CMs (*n* = 3). **(B)** Volcano plot with yellow points representing upregulated DEGs and blue points representing downregulated DEGs. Grey points represent non-DEGs. **(C)** Number of genes representing upregulated DEGs and number of genes representing downregulated DEGs for comparison combinations. **(D)** GO pathway analysis of the DEGs. The plot shows the magnitude of the expression changes (log2 fold change) vs. the statistical significance (-log10 *p*-value).

To investigate the potential interacting partners with KCNH2^G53S^, we performed transcriptomic analysis to compare control hiPSC-CMs to KCNH2^G53S^ hiPSC-CMs. RNA sequencing identified 22,822 genes in the KCNH2^G53S^ hiPSC-CMs. Among these genes, 3,857 had a fold change (FC) > 2 and a *p*-value < 0.05 and thus were considered DEGs (Figure 4A). The heatmap in Figure 4B illustrates the changes in gene expression, where increased gene expression is indicated in yellow (FC ≥ 2), and decreased gene expression is shown in blue (FC ≤ 2). Of the 3,857 DEGs, 1,358 were upregulated, and 2,499 were downregulated in the KCNH2^G53S^ hiPSC-CMs (Figure 4C). The Top 20 molecular function terms in the Gene Ontology (GO) database were identified, and the most significantly enriched terms among the modulated genes in KCNH2^G53S^ hiPSC-CMs were monoatomic ion channel activity and voltage-gated channel activity (Figure 4D). Our findings suggest that the KCNH2^G53S^ mutation affects the expression of genes involved in ion channel activity, such as KCNH2, SCN5A, and CACNA1C, as well as those regulating gated channel function, including KCNQ1, HCN4, and KCNE1. Additionally, genes associated with protein heterodimerization, such as DLG1 and CASK, exhibited altered expression, suggesting potential disruptions in intracellular protein interactions related to ion channel expression. We have provided an extended list of significantly altered genes in Supplementary Table S2 in the Results section.

### Abnormal electrophysiological profiles of KCNH2^G53S^ hiPSC-CMs

**Figure 5:**
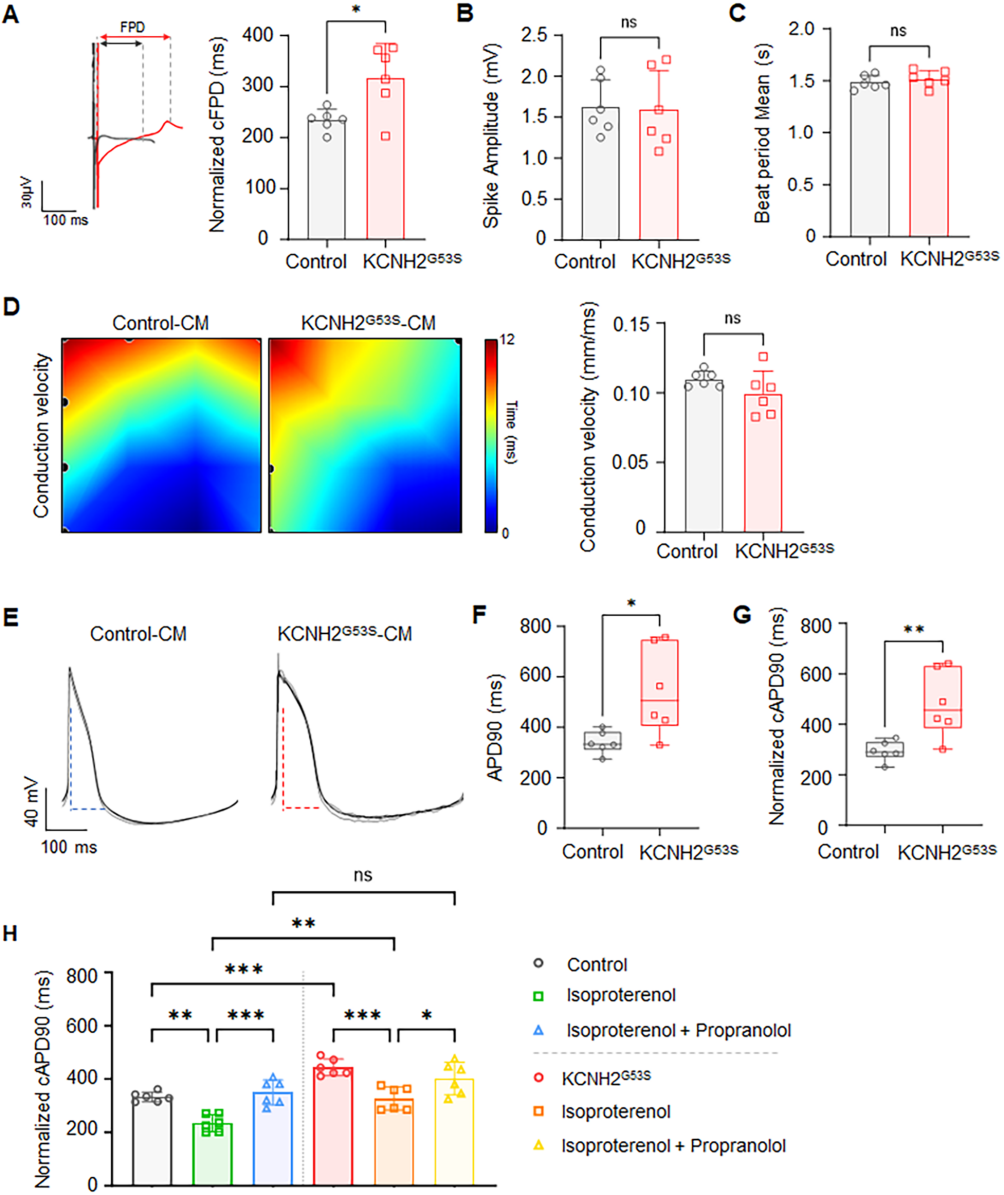
Electrophysiological profiles of control and KCNH2^G53S^ hiPSC-CMs **(A)** representative field potential tracings of control and KCNH2^G53S^ hiPSC-CMs and corrected field potential duration (cFPD) of control and KCNH2^G53S^ hiPSC-CMs recorded using a microelectrode array (MEA). **(B)** Mean beat period of control and KCNH2^G53S^ hiPSC-CMs. **(C)** Spike amplitude measurement of control and KCNH2^G53S^ hiPSC-CMs. **(D)** Conduction velocity map illustrating the propagation of electrical impulses between control and KCNH2^G53S^ hiPSC-CMs. **(E)** Representative action potential traces of control and KCNH2^G53S^ hiPSC-CMs. **(F)** Comparison of action potential duration at 90% repolarization (APD90) and **(G)** corrected action potential duration (cAPD90) of control and KCNH2^G53S^ hiPSC-CMs. **(H)** Corrected action potential duration (cAPD90) of control and KCNH2^G53S^ hiPSC-CMs treated with isoproterenol (100 nM) and propranolol (200 nM). Data are the mean ± SD. *P < 0.05, **P < 0.01.

To evaluate the electrophysiological phenotype of the KCNH2^G53S^ mutation, we characterized the electrophysiological properties of KCNH2^G53S^ hiPSC-CMs by obtaining multi-electrode array (MEA) recordings. The KCNH2^G53S^ hiPSC-CMs exhibited a significantly prolonged corrected field potential duration (cFPD), when compared to control cells (318.0 ± 66.3 ms vs. 234.5 ± 21.0 ms; *P =* 0.015) (Figure 5A). No significant difference was observed in the spike amplitude (Figure 5B) or the average beat interval (Figure 5C) between the KCNH2^G53S^ and control hiPSC-CMs. APD90 was significantly longer for KCNH2^G53S^ hiPSC-CMs than for control hiPSC-CMs (545.3 ± 176.3 ms vs. 339.9 ± 44.5 ms; *P =* 0.019) (Figure 5E, 5F), and the corrected action potential duration at 90% repolarization (cAPD90) of KCNH2^G53S^ hiPSC-CMs was also significantly longer than that of the control hiPSC-CMs (482.6 ± 133.1 ms vs. 294.0 ± 39.8 ms vs.; *P =* 0.019) (Figure 5G). Moreover, isoproterenol significantly prolonged cAPD in KCNH2^G53S^ hiPSC-CMs compared to that in control cells, but this effect was significantly reduced by isoproterenol and subsequently restored by propranolol in both mutant and control cells (Figure 5H), suggesting that propranolol may counteract adrenergic stimulation-induced electrophysiological abnormalities.

### Abnormal intracellular calcium dynamics of KCNH2^G53S^ hiPSC-CMs

**Figure 6:**
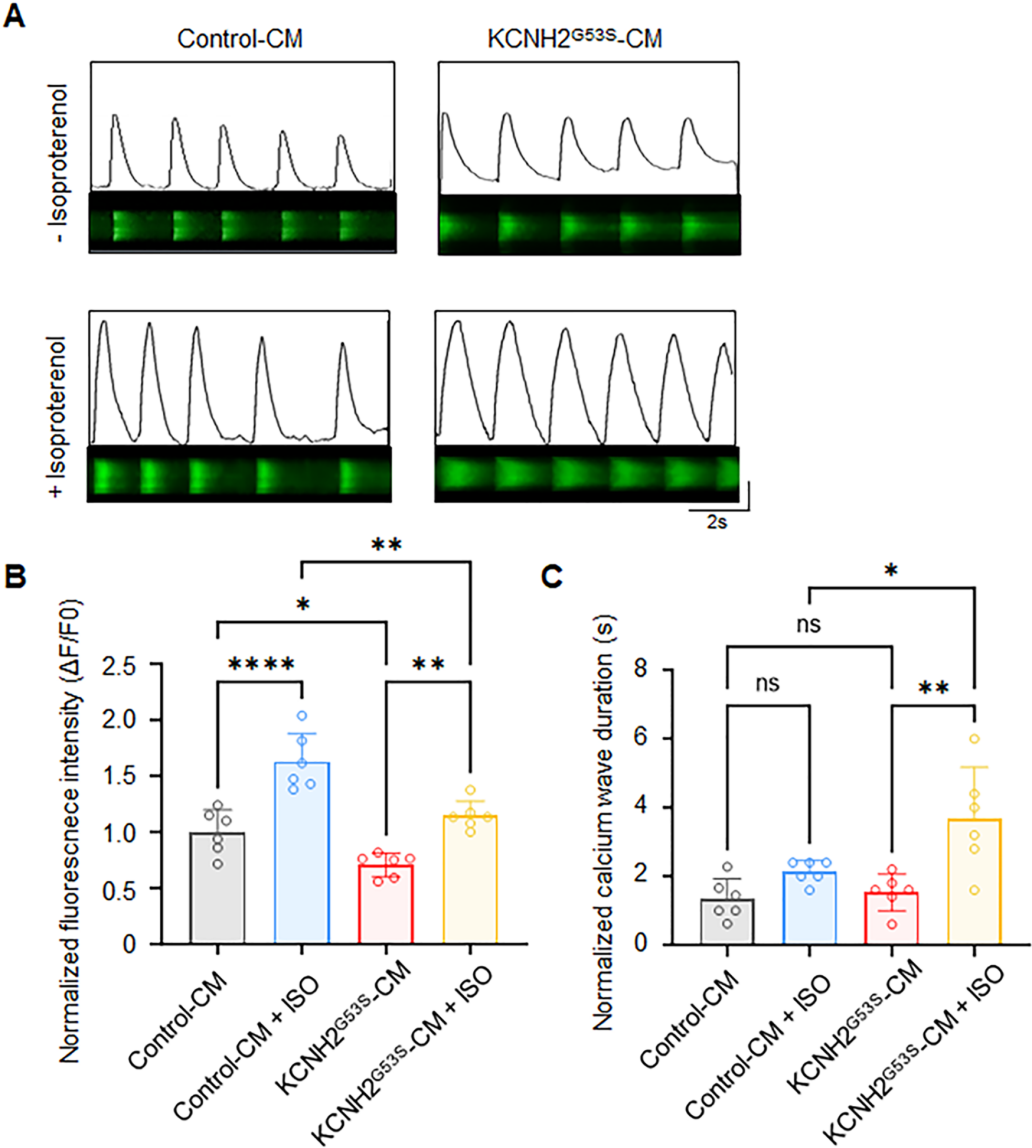
Ca^2+^ homeostasis analysis in control and KCNH2^G53S^ hiPSC-CMs **(A)** representative confocal line scans (upper) and normalized Ca^2+^ transients (lower), **(B)** normalized Ca^2+^ transient amplitude (*Δ*F/F0), and **(C)** normalized calcium wave duration from control and KCNH2-G53S cardiomyocytes. Data are the mean ± SD. *P < 0.05, **P < 0.01, ****P < 0.0001.

We investigated the intracellular calcium dynamics in KCNH2^G53S^ hiPSC-CMs using calcium confocal microscopy (Figure 6A). The results showed that the calcium transient amplitude was significantly lower in KCNH2^G53S^ hiPSC-CMs than in control cell lines under isoproterenol stimulation (Figure 6B). This increase was accompanied by a prolonged calcium wave duration (482.6 ± 133.1 ms vs. 294.0 ± 39.8 ms; *P =* 0.008), indicating exacerbated abnormal calcium handling in the presence of isoproterenol (Figure 6C).

## Discussion

In this study, we identified a heterozygous single nucleotide variant in exon 2 of KCNH2 that caused LQT2, which further underscores the importance of the PAS domain in hERG channel function. To fully realize the potential of hiPSCs for elucidating the mechanistic basis and complex pathophysiology of channelopathies, we generated hiPSC-CMs from a patient with LQT2 harboring a heterozygous mutation (c.157G>A) in the KCNH2 gene, resulting in a Gly53-to-Ser substitution within the PAS domain. This mutation was previously described in a Japanese cohort of patients with LQT2; however, its precise mechanism of action and the characteristics of patients with the KCNH2 G53S mutation have not been reported (19). Moreover, detailed studies exploring how this mutation affects ion channel dynamics, protein interactions, and downstream signaling pathways are lacking. Research on this patient is important because studying severe LQT can provide key insights into the mechanisms of cardiac arrhythmias. This study investigated the impact of a specific genetic mutation on the electrophysiological function of cardiomyocytes, which may yield information that could promote optimization of targeted therapies.

Recent research has shown that PAS domains are universal molecular sensors with plasticity, evolvability, and transmissibility, as well as potential as drug targets (20). The hERG PAS domain plays a role in mediating interactions between the domain and other parts of the channel, and is critically important for the characteristically slow deactivation of the channel (21). Understanding the impact of PAS domain mutations on channel function could provide valuable insights into the mechanisms underlying various channelopathies.

Here, we present a functional characterization of patient-specific model KCNH^G53S^ hiPSC-CM line to investigate the deep intronic KCNH2 53S variant. hiPSC-CMs generated from patients with KCNH mutations could significantly advance our understanding of the pathological mechanisms underlying LQT-related cardiac arrhythmias. Itzhaki et al. described a KCNH2 mutation that exhibited prolonged action potentials and reduced hERG currents, mimicking the clinical phenotype of LQT2 (12). Similarly, in the present study, the KCNH2^G53S^ hiPSC-CMs exhibited decreased hERG expression, explaining one possible mechanism of disease. We also identified 3,857 DEGs in KCNH2^G53S^ hiPSC-CMs; these genes were related to monoatomic ion channel activity, voltage-gated channel activity, and protein heterodimerization, indicating significant alterations in these pathways. These findings suggest that the KCNH2 G35S mutation disrupts the key pathways involved in ion channel regulation and gating, which may contribute to the pathophysiology of the disease. This study provides a functional characterization of the KCNH2 G53S variant and its association with the LQT2 phenotype while demonstrating the utility of patient-specific hiPSC-CM models in studying disease mechanisms.

In our investigation of the biophysical disease mechanisms in hiPSC-CMs, we uncovered significant findings related to the KCNH2 G53S mutation. Our findings demonstrated that KCNH2^G53S^ hiPSC-CMs displayed prolonged field and action potential durations, indicating altered electrophysiological properties. Interestingly, while spike amplitude, beat interval, and conduction velocity were unaffected, cAPD was significantly prolonged. This suggests specific disruption of the electrophysiological profile of these cells. The heterozygous the G53S mutation in the PAS domain of KCNH2 resulted in abnormal electrophysiological profiles in the hiPSC-CMs.

We also evaluated cytosolic Ca^2+^ transients as they closely reflect prolonged action potentials in hiPSC models of inherited cardiac arrhythmia (22). Brandão et al. previously described a KCNH2 mutation that resulted in significant prolongation of both Ca^2+^ transients and contraction-relaxation duration (23). Similarly, the observed abnormalities in calcium handling, including reduced calcium transient amplitude and prolonged calcium wave duration, in KCNH2^G53S^ hiPSC-CMs suggest a potential link with prolonged APD.

Therefore, considering the severity of the disease and the patient's responsiveness to propafenone treatment, the potential impact of abnormal intracellular calcium dynamics on pathogenesis, possibly influenced by APD shortening and beta-adrenergic modulation, should be considered.

A limitation of this study is that we did not generate independent iPSC clones or isogenic controls. Future studies will be necessary to determine whether the KCNH2^G53S^ mutation also impairs hERG membrane trafficking, as well as to confirm its electrophysiological impact through direct IKr current measurements via patch-clamp, which will be essential for further validation. Another limitation is the lack of direct IKr current measurements via patch-clamp, which will be essential for further electrophysiological validation in future research. Future studies should investigate how propranolol influences calcium handling and Sarcoplasmic reticulum (SR) calcium load data in KCNH2^G53S^ iPSC-CMs. Additionally, exploring the effects of flecainide in KCNH2^G53S^ iPSC-CMs could provide a more comprehensive pharmacological assessment. In terms of transcriptomic analysis, we applied a standard |Fold Change| ≥ 2 with *p* < 0.05 for DEG selection instead of multiple comparison corrections. While this approach prioritizes biologically relevant genes, future studies incorporating FDR-adjusted thresholds in larger datasets may further enhance statistical rigor.

## Conclusions

This study demonstrates that the KCNH2 G53S mutation in the PAS domain leads to impaired protein expression, disrupted electrophysiological profiles, and impaired calcium handling, which contribute to the pathogenesis of LQT2. This study not only reveals a novel mechanism underlying the pathogenic impact of the KCNH2 G53S mutation but also demonstrates a robust strategy for investigating other genetic cardiac disorders.

## Data Availability

The raw data supporting the conclusions of this article will be made available by the authors, without undue reservation.
