# Supplementary material for: Pathogenic KCNH2-G53S variant in the PAS domain influences the electrophysiological phenotype in long QT syndrome type 2
Source: Front Cardiovasc Med. 2025 Apr 9;12:1524909. doi: 10.3389/fcvm.2025.1524909 (PMC12014601; doi:10.3389/fcvm.2025.1524909)
Supplement: Supplementary file 1 [file Table1.docx]

Supplementary Materials for

**Elucidating of a novel pathogenic PAS domain variant in KCNH2 on type 2 long QT syndrome**

**Table S1. List of sequences of primers.**

|  | Primer Name | Sequences |
| --- | --- | --- |
| Genotyping | KCNH2 | F: AGTGGAGAATGTGGGGAAGG  R: GCTCCTACCATCTTTCCGGT |
| qRT-PCR | KCNH2 | F: ACA CGG CTG TCT TCA CAC CCT ACT  R: CTG ACC ACC TCC TCG TTG GCA TT |
